# Challenges and opportunities for cervical screening in women over the age of 50 years: a qualitative study

**DOI:** 10.3399/BJGP.2022.0036

**Published:** 2022-10-04

**Authors:** Alison Bravington, Hong Chen, Judith Dyson, Lesley Jones, Christopher Dalgliesh, Amée Bryan, Julietta Patnick, Una Macleod

**Affiliations:** Hull York Medical School, University of Hull, Kingston-Upon-Hull.; Division of Health Sciences, Warwick Medical School, University of Warwick, Warwick.; Centre for Social Care, Health and Related Research, Birmingham City University, Birmingham.; Hull York Medical School, University of Hull, Kingston-Upon-Hull.; Hull York Medical School, University of Hull, Kingston-Upon-Hull.; Department of Sport and Exercise Science, Durham University, Durham.; Nuffield Department of Population Health, University of Oxford, Oxford.; Hull York Medical School, University of Hull, Kingston-Upon-Hull.

**Keywords:** cervical screening, early detection of cancer, older women, primary care, qualitative research

## Abstract

**Background:**

Cervical cancer is a preventable disease. Cases in women age >50 years are predicted to rise by 60% in the next two decades, yet this group are less likely to attend for screening than younger women.

**Aim:**

To seek novel solutions to the challenges of cervical screening in women >50 years of age by examining practitioner and service-user experiences.

**Design and setting:**

Semi-structured interviews were conducted with 28 practitioners and 24 service users >50 years of age, recruited via UK primary care networks in Northern England in 2016–2017, to explore experiences related to cervical screening.

**Method:**

An inductive thematic analysis was conducted to explore the data.

**Results:**

Findings are presented under three key themes. The first, *exploring the barriers to successful cervical screening*, examines the influences of sexuality and early experiences of screening on attendance, and how preventive health care becomes a low priority as women age. The second, *the role of relationships*, explores how peer talk shapes attitudes towards cervical screening, how teamwork between practitioners engenders investment in cervical screening, and how interactions between service users and primary care over time can significantly affect intentions to screen. The third, *what constitutes good practice*, describes practical and sensitive approaches to screening tailored to women aged >50 years.

**Conclusion:**

Good practice involves attention to structural and practical challenges, and an understanding of the role of relationships in shaping screening intentions. Experienced practitioners adapt procedures to increase sensitivity, and balance time invested in problem solving against the benefits of reaching practice targets for attendance. Building networks of expertise across multiple practices can increase practitioner skill in screening this age group.

## INTRODUCTION

Cancer of the cervix is one of the most preventable forms of the disease, as precancerous cells can be identified using a screening test. In the UK, where routine screening commenced in 1988,^1^ it is estimated to prevent up to 3900 cases of cervical cancer and save 4500 lives each year.^2^^,^^3^ The landscape of cervical screening has undergone considerable changes in recent years. In 2004, the UK introduced liquid-based cytology, in which the cells brushed from the cervix are washed and filtered before examination. By 2008, this replaced the previous technique of smearing cells on to a slide. Alongside these changes, the discovery of the human papilloma virus (HPV) as a causal agent of cervical cancer in the 1990s^4^ led to the introduction of vaccinations in the UK against high-risk strains of this sexually transmitted infection^5^ for 12- to 18-year-old girls in 2008, and for all 12–18-year-olds by 2019. HPV became the primary screening test in 2019, with cytology as follow-up for individuals with a positive result.

The HPV vaccine is most effective if administered before a person becomes sexually active.^6^ It will be decades before the effects of vaccination are evident in reducing the incidence of cervical cancers across all age groups. The incidence of cervical cancer among women >50 years of age in the UK is predicted to rise by 62% over the next 20 years,^7^ as the first cohort of HPV-vaccinated women do not reach age 50 years until 2044; by 2036, the highest incidence of cervical cancer will be seen in women aged 50–59 years.

In the UK’s national screening programme, the frequency of testing drops from every 3 years to every 5 years at the age of 50, stopping at 64 years. Many women associate ageing with a lowering of risk^8^ and are less likely to continue screening;^9^ in the UK, a quarter of women aged 50–64 years do not attend.^10^^–^^12^ Self-HPV testing has been trialled in women of this age group, but does not appeal to all women, and a mix of approaches is likely to be the best way forwards in protecting this cohort.^13^^,^^14^

Studies considering how age influences attendance for cervical screening in the UK and Europe report that women >45 years of age are more likely to make a conscious decision to stop attending than younger women,^8^^,^^15^ and to cite past traumatic experiences of intimate medical examinations as a reason for non-attendance.^16^^–^^18^ Ageing can make screening more painful,^19^ and bring changes in body image that can increase women’s discomfort in allowing intimate areas of the body to be seen or touched by a health practitioner.^16^^,^^18^

**Table:** How this fits in

Women >50 years old are now in a higher-risk group for cervical cancer than younger women who have been vaccinated against human papilloma virus (HPV). In the UK, a quarter of women >50 years do not attend for cervical screening, and most women are still uncomfortable about self-screening for HPV. Previous qualitative studies have focused on negative emotions and risk perception among older women but have failed to explore the practical challenges of screening. This multisite study examined service-user and practitioner narratives about cervical screening in this age group, and offers recommendations for good practice.

The existing literature focuses on the physical and psychological discomforts of an invasive screening procedure, and fails to consider the wider social context surrounding the practice of screening with women aged >50 years, including practitioner perceptions of screen taking and the influence of practitioner–patient relationships. The aim of this project was to seek novel solutions to the challenges of cervical screening in women >50 years of age by examining both practitioner and service-user experiences. The study took place before the COVID-19 pandemic, a time in which face-to-face appointments in UK primary care became impossible or difficult and the problems in screening attendance addressed by this research were exacerbated.

## METHOD

### Design

In-depth, in-person semi-structured interviews with service users aged >50 years and practitioners were undertaken to explore experiences of cervical screening. This study was conducted before the COVID-19 pandemic.

### Participants and recruitment

Ten general practices in Northern England were recruited to the study in 2016–2017 across areas with a range of levels of deprivation^20^ in and around two cities, one city with a high level of ethnic diversity. All practitioners at each site with experience of cervical screening were invited to volunteer for interview. Service users aged 50–64 years were recruited purposively via GP practice lists to include regular screening attenders and non-attenders — women who had not attended for at least 1 year beyond their last screening invitation (recruitment focused on women who were several years beyond their most recent screening invitation). Participating practices posted study information to women >50 years old eligible for cervical screening, including all non-attenders (up to a maximum of 250) and randomly selected regular attenders (up to a maximum of 50) identified through a database search. Where no non-attenders volunteered for interview, practitioners undertook follow-up telephone calls to up to 10 non-attenders who had received study information. Service users who wished to volunteer responded to the GP practice, and their contact details were passed to the research team with their permission.

A sample size of 60 was prespecified, aiming for 15 interviews across each of the four perspectives relevant to the study (screening attenders/non-attenders/GPs/practice nurses), based on recommendations around reaching data saturation in 12 interviews^21^ and an understanding that evenly distributed recruitment across the four perspectives might not be possible in the time available.

### Data collection

The study was grounded in social constructionist epistemology, taking the view that our experiences are not recounted in objective and unbiased ways, but filtered through our perceptions of the world.^22^ Interviews were conducted face-to-face by a female research associate with a PhD and 10 years’ experience in applied health research. Participants knew in advance that the researcher was female and in her 50s, and that the study was funded by a registered cancer charity to investigate service-user and practitioner experiences and develop content for interventions to inform women about cervical screening.^23^

Interviews explored experiences of cervical screening tests among service users and practitioners. The interviewer probed to explore age-related challenges, attitudes towards risk (personal, and professional where appropriate), and examples of perceived ‘good practice’. (See Supplementary Boxes S1 and S2 for interview guides.) Interviews were audio-recorded, transcribed, anonymised, and analysed with participants’ written consent.

### Data analysis

Data-driven thematic analysis was conducted^24^^,^^25^ in an iterative process involving four members of the research team. In the first round of coding, four research team members each coded three transcripts inductively (12 transcripts in total), and met to develop an initial coding framework through discussion. Two research team members used this framework to code the remaining transcripts, developing further codes and refining the overarching themes in an iterative process through further discussion, until agreement was reached on a finalised framework. NVivo version 10 was used for data management. Selected data are presented (the full dataset is available from the corresponding author on reasonable request).

## RESULTS

**Figure 1.**
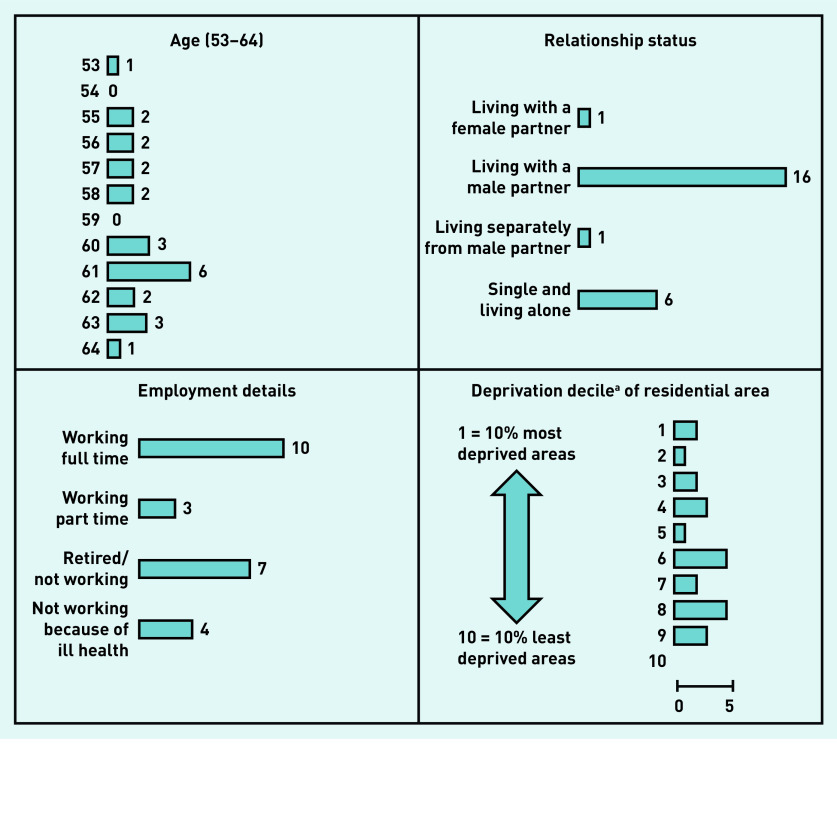
*Age in years, relationship status, and employment details of service-user interview participants, which informed their accounts of cervical screening attendance, and the deprivation decile of participants’ residential areas (recruitment spread across a range of areas).* *^a^UK government statistics on relative deprivation in small areas in England.^20^*

Interviews were conducted with 24 service users (23 at women’s homes, one at a GP practice) lasting between 28 and 68 min (average 45 min) and with 28 practitioners at their place of work lasting between 26 and 72 min (average 46 min) in the time available for the study. Figure 1 shows service users’ details; Table 1 shows research sites and practitioner details.

**Table 1.** Details of research sites and practitioner interview participants (all female)

**GP practice location and list size^a^**	**Deprivation decile^b^ of** **local area**	**Role**	

		**Practice nurses,** ***n***	**GPs,** ***n***
**Site 1: Rural town, 10 000 patients**	6	3	—

**Site 2: Town on outskirts of city, 7500 patients**	6	1	2

**Site 3: Town 5 miles from city, 12 000 patients**	6	1	2

**Site 4: Rural village, 6000 patients**	7	2	0

**Site 5: Town on outskirts of city, 8500 patients**	1	1	1

**Sites 6/7 (practitioners worked across both practices):**			
New-build area, outskirts of city, 21 500 patients	7	3	3
Urban area within city, 12 000 patients	4	—	—

**Site 8: Urban area within city, 13 000 patients**	3	3	—

**Site 9: Town 19 miles from nearest city, 17 500 patients**	9	3	1

**Site 10: Urban area within city, 3000 patients**	1	1	1

**Total**		18	10

a *Approximate list size (to the nearest 500) at the time of interview recruitment.*

b *UK government statistics on relative deprivation in small areas in England (see alsoFigure 1) with 1 indicating the most deprived areas.^20^*

All service users who volunteered for interview were White British. As interviews progressed, it became clear that some attenders had experienced periods of delayed attendance (between 2 and 10 years) that they wished to describe; these women were identified in this analysis as ‘Attender with complex story’.

Selected data are presented under three themes.

- Exploring the barriers. This examines the significance of early screening experiences, sexuality, and changes in attitudes towards preventive health care.
- The role of relationships. This explores how practitioner networking creates investment in screening women aged >50 years, and how women’s interactions with primary care and with their families shape intentions to attend.
- What constitutes good practice? This describes approaches to cervical screening that are sensitive to the needs of women >50 years of age.

**Figure 2.**
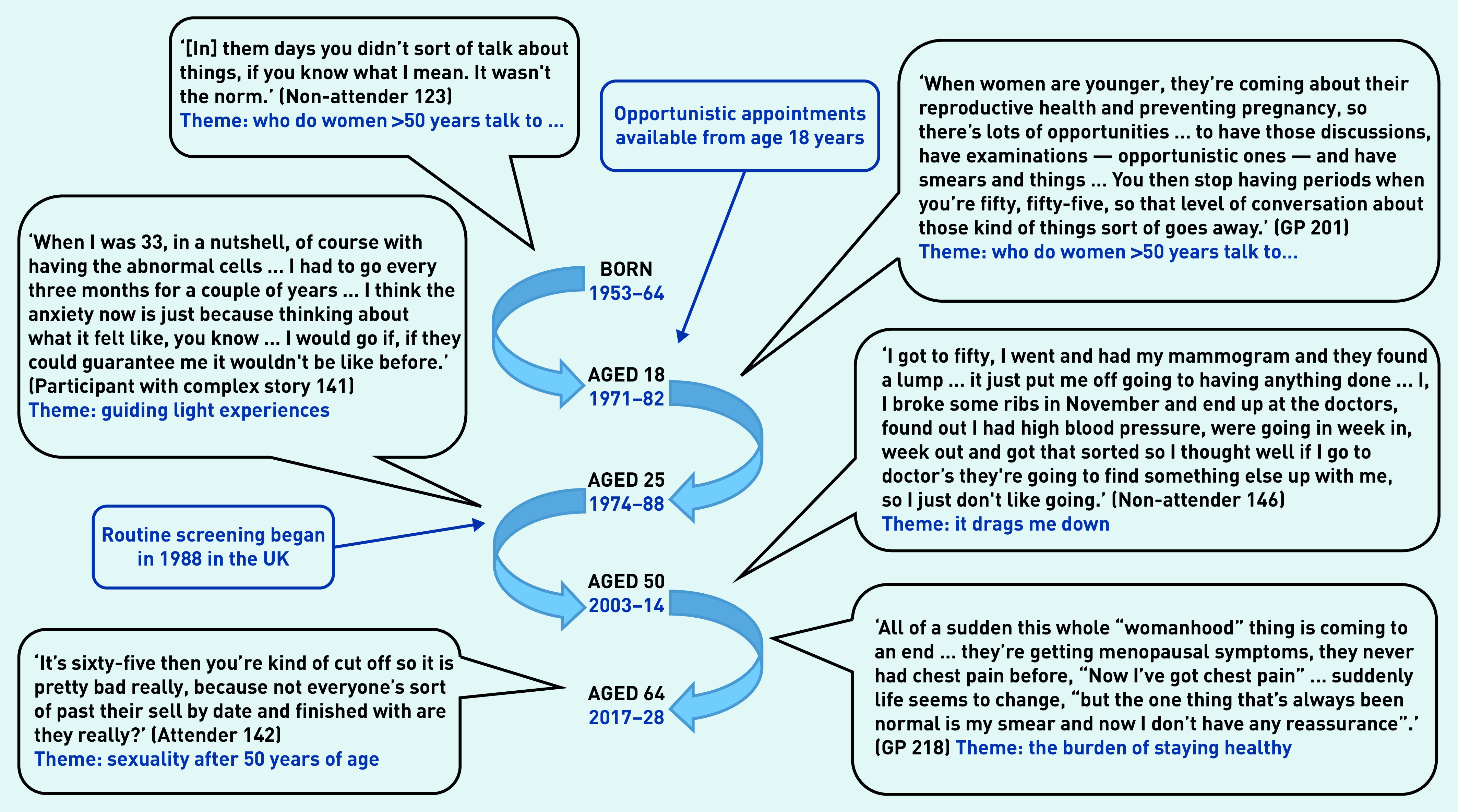
*The range of years across which service-user participants (aged 53–64 years) reached a particular age, with data quotes reflecting different stages of their life course.*

For additional qualitative data, see Supplementary Table S1. Figure 2 shows the age range of service-user interviewees over seven decades, to set their experiences in a temporal context.

### Theme 1: exploring the barriers to successful cervical screening

Barriers to successful screening emerged from experiences accumulated throughout adulthood, including the lasting significance of early experiences of screening, and changes in functionality, lifestyle, sexual partnerships, and family dynamics across the decades.

#### ‘Guiding light’ experiences

The characterisation of cervical screening as *‘a very intense kind of space’* (non-attender 102) was resonant throughout the data.

All interviewees described difficult experiences. For some, memories of early screening tests with paternalistic overtones became a significant and persistent emotional burden, resulting in an enduring antipathy to screening:

*‘It was it was like being assaulted really, it was that bad. I thought I’d picked myself a nice younger female GP … I hadn’t had sex — she never asked … I jumped off the couch half way through and I said “I’m not sure about this …” Oh, she was quite authoritative … “Just try again!” It was horrific … that’s sort of been my guiding light, that experience.’*

(Non-attender, 102)

*‘Ladies of a certain age might think to themselves it was an abusive experience, that could be a reason why some women are reluctant to go these days … I was terrified.’*

(Attender, 138)

Key features of non-attenders’ discomforts included metal speculums and a lack of rapport with practitioners. Practitioners conceptualised negative experiences as a psychological barrier with physical effects that made the insertion of a speculum difficult.

#### ‘Are you saying I’m past it?’: sexuality after 50 years of age

Sexuality was not addressed in the interview guides, but 10 service users raised this (nine with male partners, one with a female partner); five women (average age 59 years) were still sexually active with male partners. Service users described dismissive attitudes towards the discussion of sexuality and vaginal atrophy after menopause by practitioners who had affected their decision to continue screening; practitioners described difficulties discussing these issues with some service users:

*‘I had gone* [10 years ago] *, when I started with the problems after my menopause, to see a lady doctor … it wasn’t important the fact that I had no sexual intercourse … and the marriage was breaking down. And she, “Oh if that’s all that’s bothering you!”, sort of thing.’*

(Attender with complex story, 111)

*‘A lot of the time I think it’s a case of “Why? Are you saying I’m, I’m past it?” Quite a frequent expression we hear … they just feel like they get left a little bit after this.’*

(Practice nurse, 219)

Practitioners felt that changing relationship dynamics over recent decades, with the increasing acceptability of multiple intimate relationships across a lifetime, raised risk in this age group, and that women’s perceptions of risk have not caught up with this lifestyle change. Service-user data suggested that sexually active women were aware of their raised risk.

Practitioners questioned whether ending cervical screening between 59 and 64 years was appropriate. Reasons to maintain the status quo centred around the importance of supporting evidence-based guidelines, and suggestions that changing the age range may not be cost-effective or have an impact on women’s willingness to attend.

#### ‘Your view on life changes’: the burden of staying healthy

Service users and practitioners described how chronic illness and/or a lack of mobility made the conventional position for screening difficult (lying down, ankles together and knees apart). Chronic health difficulties made it difficult to predict whether an appointment booked in advance would be possible on the day, and brought fundamental changes in attitudes towards preventive health:

*‘I went through a stage I was really poorly … I thought I was dying. So your view on life changes … age is a factor, illness is a factor … you become more of a sponge to what’s going on in the world, and there’s not much you can do about dying or preventing your own death, so it becomes less important.’*

(Non-attender with multiple sclerosis, 108)

Multiple GP consultations, some of which resulted in referrals or expectations to undertake preventive health measures, were interpreted by some service users as a burden that increased with age. Women with families found themselves sandwiched between work and supporting grandchildren, adult children, and older parents; their own health was a low priority. Screening invitation letters were stockpiled, treated *‘like an overdue gas bill’*. (Attender 138):

*‘… they’ve put it in their pile of letters and the day’s gone on and they’ve forgotten, or they’ve rung up and they couldn’t get through … if that happens it can go on and on for years.’*

(Practice nurse, 217)

### Theme 2: the role of relationships

Relationships between practitioners, between practitioners and service users, and between service users and family members, had a fundamental influence on screening intentions.

#### ‘Older women need to be taken care of’: matching and networking

Cervical screening was seen as having become an exclusively female practice. The majority of service users preferred to be screened by women; practitioners felt that this influences the motivation for undertaking accreditation (which requires 12 hours of cervical screening training, 20 opportunities to take an acceptable sample, and a clinical assessment).^26^ Practitioners booking appointments in larger practices capitalised on similarities in sex and age, and established therapeutic relationships, ‘matching’ practitioner and service-user to maximise empathy in the screening encounter:

*‘It’s really hard to get appointments … if you had a relationship with the nurse then I think you probably would do that more.’*

(Attender with complex story, 141)

*‘I find that women who have a good relationship with a nurse or, or a doctor feel at that, that age, that’s the age where you want to have a relationship with who’s doing a smear, I think … older women feel that they need to be taken care of.’*

(GP, 218)

In larger practices with sufficient capacity, screening was a collective responsibility, and networking with other screen takers (for example, creating relationships with colposcopy clinics) enabled personal investment and skill sharing around screening women >50 years of age.

#### ‘It drags me down’: interactions between service users and primary care

Among non-attenders in particular, an unwillingness to engage in screening was justified by the perception of systemic difficulties in the UK NHS (pre-pandemic). The way that lifestyle choices had played out in middle age, in particular in relation to smoking and exercise, was perceived as mediating the right to access care:

*‘It’s a choice I make … GPs are there to treat people who are sick … when I do eventually go to the doctor’s I shall be bottom of the list because I’m a smoker and that’s it, my choice … even more so now the National Health Service is in such a mess.’*

(Non-attender, 143)

Self-castigation in relation to health issues was made more acute by unwelcome censure from practitioners:

*‘I feel I’m judged … Am I doing this? Am I doing that? … Bloody hell, there’s no hope for me really, is there?’*

(Non-attender, 148)

Twelve participants described the booking process as a considerable barrier, finding it stressful in person and via the telephone (*‘It all just seems a farce’* [Attender, 114]):

*‘Never mind getting the appointment, never mind actually on the bed and doing what you need to do … it is a barrier, the stress of having to check in … oh, I feel it drags me down … the whole procedure of “Reception”.’*

(Non-attender, 148)

Opportunistic booking of screening during appointments for other issues could be a double-edged sword — effective in some circumstances, but alienating if women felt disempowered:

*‘… it’s about not putting people off too much — being a bit of a conscience but not making them feel like “Can’t go and see them cause they’re gonna force me to have my smear”, or “force me to do whatever”… you’re trying to get them on board rather than being adversarial.’*

(GP, 201)

Some participants had taken to consulting pharmacists in preference to visiting their GP surgery (*‘somewhere I tend to avoid’* [Attender 114]). Booking a screening could also lead to anxiety about having to cancel (*‘I don’t want to be part of letting the system down’* [non-attender, 136]).

#### ‘I don’t discuss things like that’: who do women aged > 50 years talk to about cervical screening?

Ten service users described family health talk as a factor contributing to awareness and attendance; this was echoed in practitioner data:

*‘I don’t even discuss things like that* [cervical screening] *with my mum* [laughs] *. No, no — we’re not that sort of family.’*

(Attender with complex story, 111)

*‘If you don’t discuss sex as a, as a family between women, you may not discuss smears. So actually it becomes something that nobody really talks about … And then if nobody talks about it then nobody really sort of persuades you that it’s a good idea.’*

(GP, 201)

Taboos surrounding family talk about intimate health issues during childhood were contrasted with a deliberate openness in talking to adult children about health in the present day. Ten service-user participants had adult daughters who were too old to have benefitted from the HPV vaccination. Talk between mothers and daughters provided a forum for information exchange and encouraged screening. Of the five non-attenders who talked about family relationships, none had experience of talking to female family members about screening.

Outside of the family, mammograms were a more prevalent source of discussion with friends than cervical screening. The cervix was seen as hidden and private — *‘out of sight, out of mind’* (Non-attender, 149) — and only talked about if abnormalities occurred.

### Theme 3: what constitutes good practice?

Practitioners with extensive knowledge around the effects of menopause adjusted their approach to screening by prioritising ‘history taking’ (listening to women’s stories about sexual activity and intimate clinical examinations), prioritising step-by-step consultations and practical problem solving.

#### ‘Ask the question’: history taking as the key to successful screening

Asking *‘Why* don’t you attend? *’* and addressing problems facilitated attendance. Non-attenders had not been asked, and actively wished to discuss their decision; service users with complex stories described how addressing concerns led to the resumption of screening:

*‘… when I didn’t attend they never asked me why … That is nearly ten years. Ten years. Nobody had said … until I saw this one particular lady doctor, “Why haven’t you had it done?” And with that I burst into tears and told her all my worries and she said “Oh, we can sort that out.”’*

(Attender with complex story, 111)

*‘Ask the question. So remind them first of all that they need it and then ask them the “Why”… and be prepared to do something about it … you may not be able to just pass it on to somebody else.’*

(GP, 201)

Practitioners who believed in the centrality of history taking to successful screening in women >50 years old championed multiple consultations, feeling that this was a worthwhile investment of time:

*‘You may need to be able to take time across multiple consultations to get there. But it’s about the ultimate aim and not about … getting it this time but then stopping them ever wanting another one because it’s so traumatic.’*

(GP, 201)

#### ‘Learn the tricks’: practical solutions

Where GP practices enabled skill sharing, practical hints and tips for screening older women were passed between colleagues — *‘you get to learn the tricks.’* (Practice nurse, 207).

Some practitioners prescribed diazepam to ease anxiety, but the key practical solutions for this age group addressed mobility issues and vaginal dryness. Alternative positions such as lying on one side on the screening couch, or placing feet on the practitioner’s shoulders, could make screening possible for women with mobility problems:

*‘Maybe you just need to be a little bit more innovative about how we approach things … difficult smear does not have to equate to no smear.’*

(GP, 218)

Service users who experienced pain during screening because of dryness felt that some practitioners misinterpreted this as a failure to relax — a misunderstanding that damaged trust and rapport.

*‘I had a bad experience, just after I was fifty. I went through quite an early menopause, and then — do they call it vaginal atrophy? … I went for my smear test, the lady that did it wasn’t very sympathetic and it was awful … she said it was my fault because I wasn’t relaxed … I was very, very sore. I was very, very upset … I thought in five years … I’ll have got over it, but when the five years came I just didn’t go back.’*

(Attender with complex story, 111)

Dryness was addressed by using the smallest speculum possible, warmed with water, with a small amount of lubrication on the shaft. Some practitioners prescribed topical oestrogen cream or pessaries for 4–6 weeks before screening.

## DISCUSSION

### Summary

Three top-level themes characterise the data in this study, focusing on exploring barriers to attendance for screening in women >50 years of age, the role of relationships in encouraging screening, and what good screening practice might look like for this cohort. Barriers evolved over decades, and persisted if left unacknowledged. Family member and practitioner communication played a key role in shaping screening intentions. Good practice hinged around two issues: a willingness to ask non-attenders why they do not attend and active problem solving. The crucial resource was the investment of time in encouraging the transition from non-attender to attender.

### Strengths and limitations

The strength of this study lies in its focus on the practice of screening, and its consideration of how practitioner and service-user perspectives might be integrated to form a picture of ‘good practice’.

The study was not able to address the broad range of cultural diversity in screening responses^15^^,^^27^ or barriers to screening related to sexuality or sex^28^^,^^29^ evident in the broader literature. Only 53 participants reached interview in the 10 months available for data collection. Despite recruitment across two urban locations in Northern England with diverse demographics, all service-user volunteers for interview were White British women; only one service-user was in a same-sex relationship. As a result, the cultural and social norms arising in the data cannot be considered representative of all those aged >50 years who are eligible for cervical screening.

Minimal data on the relationship between cultural and religious frameworks and difficulties with screening attendance suggested that further exploratory qualitative research focusing on culturally specific groups in relation to gynaecological health over the age of 50 years is imperative.

### Comparison with existing literature

Changes in health and functionality can impair preventive health behaviour as people age.^30^ Research demonstrates additional concerns surrounding cervical screening, with women >50 years old increasingly at risk over the next two decades. Women who decide not to take part in screening tend to be older,^9^^,^^16^^,^^17^ and embarrassment and pain during screening are experiences shared across all ages^18^^,^^27^ but become more significant after menopause. The literature reports a divergence in service-user views about the relevance of cervical screening after the age of 50 years, with some women feeling more vulnerable and others feeling that their risk declines.^27^

Existing literature referencing practitioner experiences focuses on capturing service users’ attitudes^17^^,^^18^ or experiences of screening younger women.^16^ To the authors’ knowledge, practical advice on making the screening encounter more sensitive to the needs of women after menopause is lacking. The current study focused on service-user and practitioner accounts of cervical screening in women age >50 years. The findings demonstrate that many women in this cohort experience burgeoning family responsibilities and changing relationship patterns as they age.

The lack of acknowledgement of older women’s sexual problems by some practitioners is a barrier to continued attendance in this cohort, and the normalisation or dismissal of these issues work against intentions to attend.^31^ Addressing barriers through history taking and adjusting techniques during the screening encounter can encourage willingness to undertake or recommence screening. Networking among screening and colposcopy practitioners can enable skill sharing focused on creating and sustaining these intentions.

For the women in this study, family responsibilities — a barrier to attendance more usually associated with younger women^16^ — now stretched across four generations, from older parents to adult children and grandchildren. These findings reflect complexities highlighted in sociological literature on cervical screening.^32^^,^^33^ The prioritisation of personal health in this cohort was further compromised by changes in their attitude towards the healthcare system over time. Accessing GP appointments could become an uncomfortable procedure, complicated by perceptions of limited resources. Symptomatic and diagnosed illnesses were construed as appropriate grounds for consultation, but preventive health was linked to lifestyle choice.

Good relationships with practitioners are known to increase service users’ self-efficacy and understanding of screening.^34^ The findings in the current study suggest a central role for practitioner–patient relationships. The data support the literature reporting a preference for female cervical screening practitioners,^35^^,^^36^ and demonstrates that, as people age, experiences of screening become more strongly shaped by the quality of the interaction, and by continuity of care. Practice nurses are underutilised as a force for behaviour change — they are often willing to discuss their own lifestyle choices with patients to facilitate communication around risk factors,^35^ and are well placed to provide sensitive preventive care. Peer-topeer communication is also well recognised for its interrelationship with health^37^ and screening,^38^ and is a process often co-opted into the implementation process in cervical screening interventions outside of the UK.^39^^–^^41^ For the service users in this study, outside of the GP surgery, cervical screening was usually broached only within close relationships. Where family, work, or wider social networks were smaller, social influences on screening decisions were reduced.

### Implications for research and practice

The issue of cervical screening in women aged >50 years demands attention, given the likely increase in cervical cancer incidence in this age group over the next two decades, combined with the effects of the pandemic on face-to-face appointments. A recent trial of non-speculum HPV home testing demonstrated that not all women feel confident to self-sample, and that conventional screening attendance, although higher in the 4 months after the intervention, was similar across 12 months.^13^^,^^14^ It is likely that a combination of solutions is required.

Research addressing service user experiences would benefit from considering examples of good practice alongside exploring the challenges of service provision. Time invested by practitioners in exploring reasons for non-attendance, although often dependent on capacity, can better serve this cohort and help meet subsequent practice targets for screening (the UK incentivises goals for attendance through the Quality and Outcomes Framework).^42^ Screen taking can be adapted to take into account the effects of menopause, mobility problems, and chronic illness on the body, sexuality, and relationships. Stage-by-stage consultations can kick-start attendance among habitual non-attenders. In larger group practices, building networks of expertise across multiple practice sites can increase skill sharing around these issues. Cervical screening can be usefully construed as a transaction between practitioners and service users with common interests, and drawing on shared issues related to sex and age can also encourage rapport.
